# Intellectual Disabilities Behavior Under the Lens of Embodied Cognition Approaches

**DOI:** 10.3389/fpsyg.2021.620083

**Published:** 2021-07-12

**Authors:** J. Walter Tolentino-Castro, Markus Raab

**Affiliations:** ^1^Department of Performance Psychology, Institute of Psychology, German Sport University Cologne, Cologne, Germany; ^2^School of Arts, Sciences and Humanities, University of São Paulo, São Paulo, Brazil; ^3^School of Applied Sciences, London South Bank University, London, United Kingdom

**Keywords:** embodied cognition, mental interventions and body interventions, theoretical testability, intellectual disabilities, impaired cognition, systematic review, meta analysis

## Introduction

Diverse empirical studies have examined particularities of atypical behavior of the intellectually disabled (ID) population, and just a few theoretical approaches have been empirically tested to further understand the reasons for such atypical behavior (see Berghs et al., 2016, for medical, human rights, and social views about this topic). It is surprising that most of the theoretical approaches tested stem from research with typically developed humans, and have been adapted to partially fit the population in focus here (Bukow, 2013). For instance, Just et al. (2012), Sinha et al. (2014) share a more neuroanatomic view to explain the particularities of atypical behavior, claiming that this population lacks structural and functional body abilities in comparison with typically developed humans (Kaplan et al., 1998). More precisely, it is claimed that the misfunction of specific brain areas are the key elements for their atypical behavior. Indeed, scientific findings have reported mechanisms in which the mentioned neuroanatomic peculiarities impact their cognitive development and vice-versa; which is assumed to guide human behavior (Dye and Pascalis, 2017). Thus, to extend the traditional view a new view on embodied cognition (EC) approaches will explain atypical behavior of the intellectually disabled population (Shapiro, 2011). These approaches claim that body sensorimotor experience is the core stone of cognitive and behavioral development. The discussion though will cover the topic of whether EC approaches can be used to further enlighten the understanding of particularities of atypical behavior of individuals with IDs.

## Intellectual Disabilities Under the Lens of Embodied Cognition Approaches

EC approaches set a new era of cognitive science. It has been claimed that EC describes some of the most complex phenomena of human cognition and behavior, through conceptualization, replacement, and constitution (Shapiro, 2011, p. 9). In more details, *conceptualization* describes that the properties of an organism's body limit or constrain the concepts an organism can acquire, *replacement* states that an organism's body in interaction with its environment replaces the need for representational processes thought to have been at the core of cognition; and *constitution* claims that the body or world plays a constitutive rather than a merely causal role in cognitive processing (Shapiro, 2011). Albeit the explanation from these approaches may cover numerous examples of human behavior, criticism has been advocated recently (Bukow, 2013; Ionescu and Vasc, 2014), given EC may not (equally) well predict behavior for all sorts of human kinds (Shapiro, 2011, p. 90). At least for those with disabilities, which represent 15% of the worldwide population (WHO, 2011), we want to test the generalizability of the EC claim to the ID population. We argue that the theoretical debate on embodied cognition and the existing scientific evidence in multiple fields and populations indicates that a test of generalizability of EC to the ID population is warranted. Theoretically, embodied cognition approach proposes that abstract concepts are grounded in concrete concepts that can be perceived with our sensorimotor system. It is assumed that the abstract concept of time is based on the concrete concept of space: This is reflected, among other things, in our language: “The evening lies before me” is a sentence with temporal information that is expressed with a spatial expression “before” (Lakoff and Johnson, 1999, p. 34). Empirically, multiple populations of different sensorimotor experiences over the life span have been tested indicating robust evidence in multiple domains and tasks (Löffler et al., 2016).

What follows is guided by two arguments:

*First*, we discuss the challenges of considering the existent stratum of EC approaches (Smith and Gasser, 2005) to understand the atypical behavior of the mentioned population (Just et al., 2012; De Jaegher, 2013; Franceschini et al., 2017). Note, EC considers the body the center of individuals' experiences to produce a behavior, thus based on EC assumptions an atypical behavior could be a mismatch of environmental information processing and the experience perceived. In other words, given EC claims the relationship with the environment is mandatory to create experiences, any missing information might impact behavior. For instance, an under-estimation or over-estimation of the size of the first stair may lead to a dangerous upwards walk of stairs.

*Second*, we constrain our discussion to atypical behavior to those found in empirical studies; more precisely, assessments made via reliable motor tests in comparison to peers with typical development. For instance, recent literature has shown that persons with IDs perform poorly at motor and cognitive battery tests (Hartman et al., 2010; Westendorp et al., 2011; Houwen et al., 2016). Consequently, as hypothesized by EC approaches, poor motor achievement in motor tests has been claimed to impact cognitive development of persons with IDs, similarly well as for those with typical development (Hartman et al., 2010).

Furthermore, some authors share the view about a tight link between cognition and behavior in this population (De Jaegher, 2013; Hamilton, 2013). Although unclear how, a study suggests the reasons for this population's atypical behavior is based on the known impaired cognitive skills of this population (Lott and Dierssen, 2010). A neuroscientific perspective may describe such suggestion to stem from an abnormal functioning and structural architecture of the brain, as key factors to drive peculiarities in this population's behavior. Although scarce, some pieces of evidence support this perspective's claim (Bartlo and Klein, 2011; Hötting and Röder, 2013).

In extension to neuroscientific perspectives, the persons' experiences that are restricted have been debated. For instance, in a systematic review socialization has been reported as the most prominent source for observed atypical behavior of this population (Hamilton, 2013). In addition, recent empirical evidence suggests that the restricted motor abilities may explain partially the isolated social behavior of persons with autism (De Jaegher, 2013; Sinha et al., 2014). Sinha et al. (2014) reported that adults and children with autism present impaired capacity to predict the next (future) events, e.g., objects and persons that are moving, and thus this may impact directly the development in social groups. It is reported that the avoidance of such social confrontations for those with autism tends to be solved by the use of repetitive motor behavior (Sinha et al., 2014). In the same vein, Tolentino-Castro et al. (2017) and Riddell et al. (2017) extend these findings and report that participants with IDs present an incapacity to recognize other motor behavior patterns and velocities in comparison to the typically developed participants.

The process of deciphering environmental information demand is necessary to generate spatio-temporal representations, which are mandatory to create event predictions (Shapiro, 2011). Noteworthy, findings from experimental studies (Recanzone, 2009) state that preserved “channels” (e.g., eyes and ears) are essential to deciphering physical environmental information demands (e.g., sound, light, texture, vibration) and that these sensory inputs are less development in the ID population due to restricted sensorimotor experiences. We argue that it seems that the ID population presents impairments to process and use environmental information to generate sensorimotor interaction with the natural and human environment. In other words, taking sensorimotor experience as a complex interlink between the perception of the world and motor output, as predicted by EC approaches, it follows that ID have either less or incomplete (processed) sensory information and atypical motor behavior need to be explained within a joint EC approach (Dye and Pascalis, 2017). How good can such an alternative perspective explain atypical behavior of individuals with IDs? We argue this needs an empirical test to show that beyond brain abnormalities on cognitive and behavioral development sensorimotor experiences explain behavior and may challenge or extend therapeutic interventions (Roubertoux and Carlier, 2007; Enea-Drapeau et al., 2017).

## Is There A Way to Change an Explanation About the Behavior of the ID Population?

A key problem of the popular thinking and the literature regarding this topic is the claim that:

1. Not much or no considerable behavioral change can be achieved in this population (Hamilton, 2013).
2. No cognitive development is possible for those with IDs (Enea-Drapeau et al., 2017).

We argue that those claims do not reflect the current state of scientific findings (Molina-García and Vived, 2004; Kozulin et al., 2010). Kozulin et al. (2010) in line with Molina-García and Vived (2004) report that to some extent this population is able to have cognitive improvement; for instance, individuals diagnosed with Down syndrome and developmentally disabled participants.

The question which arrives is, which knowledge exit about effective changes in ID's atypical behavior and how does it impact current therapeutics, training, and interventions for this population? Table 1 was tailored to address this aim. It contains the 20 most cited reviews (systematic reviews or meta-analyses) regarding interventions for the intellectual disabled population. More precisely, we've selected the 10 most cited reviews which used body interventions, for instance: sport, physical activities and/or gymnastic; and 10 most cited reviews which used mental interventions, for instance: mental training, behavioral training and/or psychological therapy. Noteworthy is that the motivation to split the search in physical and mental training is based on the absence of any meta-analyses and systematic reviews that allow to describe moderators of both specific training regimes. Under the lens of EC, this might be mandatory because cognitive and behavioral development is a product of the interaction between person and environment. The search preferred reporting items (see Appendix 1) for systematic reviews and meta-analyses (PRISMA) published in the last 10 years (2010 onwards) for systematic reviews and meta-analyses, has been used for reporting rapid reviews, see Table 1.

**Table 1:** Overview of the 10 most cited reviews in regards to the topics body and mental interventions for intellectual disabled population.

**Section BODY**										
Authors	Bondár et al. (2020)	Fonzo et al. (2020)	Ruiz-González et al. (2019)	Kapsal et al. (2019)	Maïano et al. (2019)	May et al. (2019)	Paul et al. (2019)	Harris et al. (2015)	Ogg-Groenendaal et al. (2014)	Li et al. (2013)
Number of studies reviewed	13	22	27	109	14	19	19	6	20	10
Number of studies based on any theoretical background	7	Not reported	Not reported	Not reported	Not reported	Not reported	Not reported	Not reported	Not reported	5
Number of participants	598	54	842	4200	464	521	1331	178	91	349
% of Gender (female/male/^*^)	Not reported	Not reported	60.1% male 39.9% women	Not reported	57.3% boys (children studies), 51.7% men (adult studies)	Not reported	Not reported	Not reported	34 male 9 female 48^*^	“In general, there were more male than female participants”
% of Body interventions used	85% of the eligible studies described as PA	9.1% applied behavior analysis 4.5% conductive education 4.5% environmental enrichment 18.2% traditional physical therapy with or without aids, 9.1% hydrotherapy 9.1% treadmill 13.6% music therapy 27.3% computerized systems 4.5% sensory-based treatment	18.52% aerobic training 29.63% resistance training 22.22% mixed training 7.4% balance training 7.4% vibration PA 7.4% early stimulation 7.4% Technical aid	23.2% aerobic PA 7.1% RE training PA 27.1% move skills PA 35.5% general PA/PE 6.5% based on balance or core stability	21.4% balance and/or strength exercises 7.1% adapted play training 7.1% handball techniques 7.1% compute games 7.1% therapeutic sensorimotor training 7.1% physical development training 7.1% intensive motor skills training 7.1% physical therapy 7.1% vestibular stimulation exercises	100% Dance	5.3% Judo 47.4% TM 10.5% weight training 5.3% bike 10.5% Wii e-sports gaming	16.6% bicycle ergometer 16.6% strength and endurance training 16.6% plyometric jumps training 16.6% whole body vibration and isometric exercise 16.6% treadmill ergometer 16.6% rowing ergometer	40% walking/jogging, 15% aerobic exercise 10% football 5% dance 5% basketball 5% calisthenics 5% roller skating 15% general motor training	10% Treadmill TP 10% bicycle TP 10% rowing ergometer TP 20% progressive resistance TP 10% combined TP with treadmill and game-like exercise 10% combined TP with progressive resistance training and balance exercise 20% combined TP with cardiovascular and strength exercise, 10% weight-bearing exercise TP
**Section MENTAL**								
Authors	Surley and Dagnan (2019)	Patterson et al. (2019)	Cooney et al. (2018)	Stott et al. (2017)	McNair et al. (2017)	Hellenbach et al. (2015)	Ali et al. (2015)	Vereenooghe and Langdon (2013)	Hwang and Kearney (2013)	Nicoll et al. (2013)
Number of studies reviewed	23	20	18	12	7	4	6	22	19	12
Number of studies based on any theoretical background	23	Not reported	18	6	Not reported	Not reported	Not reported	Not reported	Not reported	12
Number of participants	319	109	798	554	89	72	309	847	Not reported	315
% of Gender (female/male/^*^)	Not reported	Not reported	Not reported	ID	Not reported	ID	Not reported in all studies	Not reported	Not reported	74.9% male 25.1% female
% of Mental interventions used	91.3% general CBT 8.7% manualized CBT	45% mindfulness-based approach 30% DBT 15% CFT 10% ACT	44.5% CBT with multiple cognitive therapy skills, 44.5% abilities to recognize emotions 5.5% cognitive mediation 5.5% abilities to access beliefs alone in relation to events in which they experienced anger	100% general CBT	35% DBT 20% individual therapy 45% different types of personal consult	50% education program 50% relaxation treatment and anger	44% anger management 14% one individual therapy and two group-based 14% relaxation 14% mindfulness based on meditation 14% problem solving and assertiveness training	82% general CBT 9% group-based psychotherapy 9% other individual psychotherapy	53% meditation 21% mindful observation of thoughts, feeling or food 11% body, thoughts or food awareness 5% CBT 5% Intention	100% general CBT

*PA, Physical Activity; PE, Physical Education; TP, Training Program; ACT, Acceptance and Commitment Therapy; CBT, Cognitive Behavioral Therapy; CFT, Compassion Focused Therapy; DBT, Dialetical Behavioral Therapy; ID, Incongruous description; TM, Treadmill; RE, Resistance;*

* *not reported*.

We showed above that not much empirical studies with this population have been driven by a theoretical approach. Especially, none of those scientific studies has tested/falsified EC approaches assumptions. In addition, in Table 1 (see section BODY) the majority of the intervention was performed individually (see row “*% of Body interventions used*”) and we propose to have group interventions to enhance sensorimotor learning by movement observation and sensorimotor social interactions. We can conclude that instead of using therapeutic interventions to control for the social weakness of this population, it may reinforce the social isolation between the peers and reduces potential development. In regards to Table 1 (see section MENTAL) it seems that the therapeutic interventions were driven more to avoid or minimize further other comorbid atypical behavior such as fear, anger, and sexual aggression behaviors. The fact that only a few interventions were motivated by a theoretical assumption may lead to a less evidence-based routine of practitioners and may not allow innovation in intervention strategies. Such a theory-practice gap seems to be evident in many graduate courses of psychology, physiotherapy, sports science, or medicine that often fail to combine theoretical models for typically developed humans and test how to generalize them to individuals with special needs. Likewise, most empirical evidence in the EC perspective has been conducted in the normal student population and thus generalizability is open to future research opportunities. The main idea of Table 1 is to give an overview about the current empirical evidence in this field by presenting the 10 most cited reviews. We believe that the table might help the reader to understand how our opinion is based in a systematic description of existing reviews.

## Research Opportunities

Given that experimental studies reported in Table 1 suggested diverse positive effect on cognitive and behavioral development for those with IDs, either for interventions focused in the physical or mental training, the follow research opportunities could be considered. First, follow-up studies should test whether “intervention embedded in EC approaches” (Dandashi et al., 2015) have stronger effect on participants' cognitive and behavioral development than the interventions focusing purely on physical or mental training. Second, by comparing embodied cognition and non-embodied cognition interventions future research will be able to quantify and specify the effects of interventions in ID populations.

Finally, in regard to our main aim, we state the valuable impact of EC approaches to explain atypical behavior in the ID population is an opinion that deserves empirical evidence. However, we do not know the full picture of the underlying mechanisms involved in ID's atypical behavior and moderators such as kind or level of disabilities. A test of the null hypothesis of having no positive acute effect of “intervention embedded in EC approaches” compared to currently used interventions against a hypothesis that a larger change of atypical behavior in ID's can be achieved by EC interventions is an empirical question. A few researchers started to investigate sensorimotor training interventions in ID for chronic effects of longer duration (Dandashi et al., 2015). As argued above, it's an empirical test that is open for validation. In this opinion we argue that cognitive and behavioral development driven by interventions can be supported and profit from EC approaches in person with IDs. Are you ready to take this opinion to an empirical test?

## Author Contributions

The authors confirm being the sole contributor of their work and have approved it for publication.

## Conflict of Interest

The authors declare that the research was conducted in the absence of any commercial or financial relationships that could be construed as a potential conflict of interest.
