# Supplementary material for: Intellectual Disabilities Behavior Under the Lens of Embodied Cognition Approaches
Source: Front Psychol. 2021 Jul 12;12:620083. doi: 10.3389/fpsyg.2021.620083 (PMC8311120; doi:10.3389/fpsyg.2021.620083)
Supplement: Supplementary file 1 [file Data_Sheet_1.docx]

Appendix: **Intellectual Disabilities behavior under the lens of Embodied Cognition approaches**

Overview – Methods for Search Terms for:

**Sections BODY and MENTAL of Table 1**

PRISMA – rapid review protocol

01 -Search Terms

| **Main term** | **MeSH** | **Additional terms** |
| --- | --- | --- |
| Intellectual Disability | Disabilities, Intellectual; Intellectual Disabilities; Retardation, Mental; Intellectual Development Disorder; Development Disorder, Intellectual; Development Disorders, Intellectual; Disorder, Intellectual Development; Disorders, Intellectual Development; Intellectual Development Disorders; Mental Retardation; Disability, Intellectual; Idiocy; Mental Retardation, Psychosocial; Mental Retardations, Psychosocial; Psychosocial Mental Retardation; Psychosocial Mental Retardations; Retardation, Psychosocial Mental; Retardations, Psychosocial Mental; Deficiency, Mental; Deficiencies, Mental; Mental Deficiencies; Mental Deficiency |  |
| Mental Training |  | Cognitive Training |
| Cognitive Behavioral Therapy | Behavioral Therapies, Cognitive; Behavioral Therapy, Cognitive; Cognitive Behavioral Therapies; Therapies, Cognitive Behavioral;  Therapy, Cognitive Behavioral; Therapy, Cognitive Behavior; Cognitive Behavior Therapy; Cognitive Therapy; Behavior Therapy, Cognitive; Behavior Therapies, Cognitive; Cognitive Behavior Therapies; Therapies, Cognitive Behavior; Cognitive Psychotherapy; Cognitive Psychotherapies; Psychotherapies, Cognitive; Psychotherapy, Cognitive; Therapy, Cognitive; Cognitive Therapies; Therapies, Cognitive; Cognition Therapy; Therapy, Cognition; Cognition Therapies; Therapies, Cognition |  |
| Sport Intervention |  | Physical Activity Intervention |
| Exercise | Exercises; Physical Activity; Activities, Physical; Activity, Physical; Physical Activities; Exercise, Physical; Exercises, Physical; Physical Exercise; Physical Exercises; Acute Exercise; Acute Exercises; Exercise, Acute; Exercises, Acute; Exercise, Isometric; Exercises, Isometric; Isometric Exercises; Isometric Exercise; Exercise, Aerobic; Aerobic Exercise; Aerobic Exercises; Exercises, Aerobic; Exercise Training; Exercise Trainings; Training, Exercise; Trainings, Exercise | - |

02 - Data search for Intellectual Disability and Physical Activity interventions

| **Base** | | **Terms** | **n** |
| --- | --- | --- | --- |
| Pubmed | #1 | "Intellectual Disability"[MeSH Terms] | 96,364 |
|  | #2 | "Exercise"[MeSH Terms] OR "sport intervention"[All Fields] OR "physical activity intervention"[All Fields] | 199,112 |
|  | #3 | #1 AND #2:  "Intellectual Disability"[MeSH Terms] AND ("Exercise"[MeSH Terms] OR ("sport intervention"[All Fields] OR "physical activity intervention"[All Fields])) | 540 |
|  | **#4** | **#3 with Filters: Meta-Analysis, Systematic Review** | **36** |
| Web of Science | #’ | TS=("Intellectual Disability") OR TS=("Disabilities, Intellectual") OR TS=("Intellectual Disabilities") OR TS=("Retardation, Mental") OR TS=("Intellectual Development Disorder") OR TS=("Development Disorder, Intellectual") OR TS=("Development Disorders, Intellectual") OR TS=("Disorder, Intellectual Development") OR TS=("Disorders, Intellectual Development") OR TS=("Intellectual Development Disorders") OR TS=("Mental Retardation") OR TS=("Disability, Intellectual") OR TS=(Idiocy) OR TS=("Mental Retardation, Psychosocial") OR TS=("Mental Retardations, Psychosocial") OR TS=("Psychosocial Mental Retardation") OR TS=("Psychosocial Mental Retardation") OR TS=("Retardation, Psychosocial Mental") OR TS=("Retardation, Psychosocial Mental") OR TS=("Deficiency, Mental") OR TS=("Deficiencies, Mental") OR TS=("Mental Deficiencies") OR TS=("Mental Deficiency") | 65,165 |
|  | #2 | TS=(Exercise) OR TS=(Exercises) OR TS=("Physical Activity") OR TS=("Activities, Physical") OR TS=("Activity, Physical") OR TS=("Physical Activities") OR TS=("Exercise, Physical") OR TS=("Exercises, Physical") OR TS=("Physical Exercise") OR TS=("Physical Exercises") OR TS=("Acute Exercise") OR TS=("Acute Exercises") OR TS=("Exercise, Acute") OR TS=("Exercises, Acute") OR TS=("Exercise, Isometric") OR TS=("Exercises, Isometric") OR TS=("Isometric Exercises") OR TS=("Isometric Exercise") OR TS=("Exercise, Aerobic") OR TS=("Aerobic Exercise") OR TS=("Aerobic Exercises") OR TS=("Exercises, Aerobic") OR TS=("Exercise Training") OR TS=("Exercise Trainings") OR TS=("Training, Exercise") OR TS=("Trainings, Exercise”) | 597,654 |
|  | #3 | #1 AND #2 | 1,356 |
|  | #4 | #3 (REVIEW) | **145** |

03 - Data search for Intellectual Disability and Mental Training interventions

| **Base** | | **Terms** | **n** |
| --- | --- | --- | --- |
| Pubmed | #1 | "Intellectual Disability"[MeSH Terms] | 96,364 |
|  | #2 | "Cognitive Behavioral Therapy"[MeSH Terms] OR "mental training"[All Fields] OR "cognitive training"[All Fields] | 31,489 |
|  | #3 | #1 AND #2:  "Intellectual Disability"[MeSH Terms] AND ("Cognitive Behavioral Therapy"[MeSH Terms] OR ("mental training"[All Fields] OR "cognitive training"[All Fields])) | 133 |
|  | **#4** | **#3 with Filters: Meta-Analysis, Systematic Review** | **13** |
| Web of Science | #1 | TS=("Intellectual Disability") OR TS=("Disabilities, Intellectual") OR TS=("Intellectual Disabilities") OR TS=("Retardation, Mental") OR TS=("Intellectual Development Disorder") OR TS=("Development Disorder, Intellectual") OR TS=("Development Disorders, Intellectual") OR TS=("Disorder, Intellectual Development") OR TS=("Disorders, Intellectual Development") OR TS=("Intellectual Development Disorders") OR TS=("Mental Retardation") OR TS=("Disability, Intellectual") OR TS=(Idiocy) OR TS=("Mental Retardation, Psychosocial") OR TS=("Mental Retardations, Psychosocial") OR TS=("Psychosocial Mental Retardation") OR TS=("Psychosocial Mental Retardations") OR TS=("Retardation, Psychosocial Mental") OR TS=("Retardations, Psychosocial Mental") OR TS=("Deficiency, Mental") OR TS=("Deficiencies, Mental") OR TS=("Mental Deficiencies") OR TS=("Mental Deficiency") | 65,165 |
|  | #2 | TS=("Cognitive Behavioral Therapy") OR TS=("Behavioral Therapies, Cognitive") OR TS=("Behavioral Therapy, Cognitive") OR TS=("Cognitive Behavioral Therapies") OR TS=("Therapies, Cognitive Behavioral") OR TS=("Therapy, Cognitive Behavioral") OR TS=("Therapy, Cognitive Behavior") OR TS=("Cognitive Behavior Therapy") OR TS=("Cognitive Therapy") OR TS=("Behavior Therapy, Cognitive") OR TS=("Behavior Therapies, Cognitive") OR TS=("Cognitive Behavior Therapies") OR TS=("Therapies, Cognitive Behavior") OR TS=("Cognitive Psychotherapy") OR TS=("Cognitive Psychotherapies") OR TS=("Psychotherapies, Cognitive") OR TS=("Psychotherapy, Cognitive") OR TS=("Therapy, Cognitive") OR TS=("Cognitive Therapies") OR TS=("Therapies, Cognitive") OR TS=("Cognition Therapy") OR TS=("Therapy, Cognition") OR TS=("Cognition Therapies") OR TS=("Therapies, Cognition") OR TS=("Mental Training") OR TS=("Cognitive Training") | 37,722 |
|  | #3 | #1 AND #2 | 174 |
|  | **#4** | **#3 (REVIEW)** | **42** |
